# Prevalence of birth injuries and associated factors among newborns delivered in public hospitals Addis Ababa, Ethiopia, 2021. Crossectional study

**DOI:** 10.1371/journal.pone.0281066

**Published:** 2023-01-30

**Authors:** Esubalew Amsalu Tibebu, Kalkidan Wondwossen Desta, Feven Mulugeta Ashagre, Asegedech Asmamaw Jemberu

**Affiliations:** 1 St.Peter Specialized Hospital, Addis Ababa, Ethiopia; 2 School of Nursing and Midwifery, College of Health Science, Addis Ababa University, Addis Ababa, Ethiopia; 3 Department of Medical Laboratory Sciences, College of Health Science, Addis Ababa University, Addis Ababa, Ethiopia; Bangabandhu Sheikh Mujib Medical University (BSMMU), BANGLADESH

## Abstract

**Background:**

Birth injury is harm that a baby suffers during the entire birth process. It includes both birth asphyxia and birth trauma. In Ethiopia, birth injury has become the leading cause of neonatal morbidity and mortality, accounting around 28%-31.6% of neonatal mortality. The study aimed to assess the prevalence and factors associated with birth injuries among newborns delivered in public hospitals Addis Ababa, Ethiopia, 2021.

**Methods:**

Institution based cross-sectional study was conducted from February 15^th^ to April 20^th^, 2021 in selected public hospitals of Addis Ababa, Ethiopia. Random sampling and systematic random sampling were used. Data was entered by using Epi data version 4.0.2 and exported in to SPSS Software version 25 for analysis. Both bivariate and multivariable logistic regressions analyses were used. Finally P-value <0.05 was used to claim statistically significant.

**Result:**

The prevalence of birth injury was 24.7%. In the final model, birth asphyxia was significantly associated with the short height of the mothers (AOR = 10.7, 95% CI: 3.59–32.4), intrapartal fetal distress (AOR = 4.74, 95% CI: 1.81–12.4), cord prolapse (AOR = 7.7. 95% CI: 1.45–34.0), tight nuchal cord (AOR = 9.2. 95% CI: 4.9–35.3), birth attended by residents (AOR = 0.19, 95% CI: 0.05–0.68), male sex (AOR = 3.84, 95% CI: 1.30–11.3) and low birth weight (AOR = 5.28, 95% CI: 1.58–17.6). Whereas, birth trauma was significantly associated with gestational diabetic mellitus (AOR = 5.01, 95% CI: 1.38–18.1), prolonged duration of labor (AOR = 3.74, 95% CI: 1.52–9.20), instrumental delivery (AOR = 10.6, 95% CI: 3.45–32.7) and night time birth (AOR = 4.82, 95% CI: 1.84–12.6).

**Conclusion:**

The prevalence of birth injury among newborns has continued to increases and become life-threatening issue in the delivery and neonatal intensive care unit in the study area. Therefore, considering the prevailing factors, robust effort has to be made to optimize the quality obstetric care and follow up and emergency obstetrics team has to be strengthened to reduce the prevalence of birth injury.

## Introduction

The process of birth, whether spontaneous or assisted, is naturally traumatic for the newborns. Birth injury is the structural destruction or functional deterioration of the neonate’s body due to a traumatic event at birth [1]. Birth related injuries encompass both those due to lack of oxygen (birth asphyxia) and physical trauma during the birth process (birth trauma). Both can occur separately or in combination [2–5].

Injuries to the newborns that result from mechanical forces (i.e. compression, traction) during the birth process are classified as mechanical birth trauma. Whereas, according to the World Health Organization (WHO), birth asphyxia defined as a “failure to initiate and sustain breathing at birth” [6] It’s usually considered by low APGAR score: (Appearance, Pulse rate, Grimace, Activity and Respiration) <7 at 5^th^ minutes, arterial cord pH < 7 and base deficit >12, neonate did not cry at birth or needed resuscitation, acidosis, seizure and hypotonia [7]. Study suggested that, birth asphyxia occur due to maternal antepartum, intra-partal and post partal factors [8]. Intra-partum related factors accounts the highest proportion of risk factors for birth asphyxia (70%). Whereas, antepartum and post partal factors accounts 20% and 10% respectively [9].

According to international classification of disease 10^th^ revision (ICD-10) and different literature, the common types of birth injuries includes birth asphyxia and birth trauma (soft tissue injuries (bruises, petechial, subcutaneous fat necrosis, ulceration and perforation), extra cranial hemorrhages (cephalhaematoma, caput succedaneum, subgalial hemorrhage), intra-cranial hemorrhages, neurological injury (spinal cord injury, facial nerve palsy, brachial plexus injury such as Erb’s palsy and Klumpke’s palsy), musculoskeletal injury (long bone and clavicular fracture) [10–13].

According to 2016 WHO reports, it is estimated that 662, 000 neonatal deaths and 1.3 million stillbirths occur annually due to intra-partum related complications, or complications during labor and delivery. Birth injuries are among the three leading cause of most neonatal death worldwide which accounts for 10% of deaths in children under 5 years of age [14].

The incidence of birth injuries varies from place to place and it is mostly determined by the standard of available obstetrical management.

Birth asphyxia is a leading cause of brain damage and also survivors often experience lifelong health problems like disabilities, developmental delays, palsy, intellectual disabilities and behavioral problems [15, 16].

In developed countries, the occurrences of birth injury are decreased due to the improvements in obstetric practice and care. In Ethiopia, according to 2019 mini EDHS (Ethiopia Demographic and Health Survey) reported, the percentages of delivery by skilled providers increased from 28% in 2016 to 50% in 2019. Despite of this, the number of neonatal death increased from 29 per 1000 live births to 30 per 1000 live births in Ethiopia [17].

Reports about the prevalence of birth injures among live birth newborns are limited in Ethiopia. As far as literature review revealed that, there is a limited research done on prevalence of birth injuries among live birth delivery especially in the study area. However, intra-partum related complications among newborns during the time of delivery are still the leading cause of neonatal morbidity and mortality in Addis Ababa public hospitals. Therefore, this study was carried out to assess the prevalence of birth injuries and associated factors among newborns delivered in public hospitals Addis Ababa, Ethiopia.

## Material and methods

### Study design, study period and study area

Institutional based cross- sectional study was conducted in Addis Ababa Public Hospitals from February 15^th^ to April 20^th^, 2021. This study was carried out in four randomly selected public hospitals (Tikur Anbessa Specialized Hospital (TASH), Yekatit 12 Hospital Medical College (Y-12HMC), Gandhi Memorial Hospital (GMH) and St. Paul Hospital Millennium Medical College (SPHMMC)).

### Study population

All live birth newborns delivered in selected public hospitals with gestational age of ≥ 28 weeks were included in this study. Neonates with major congenital anomalies (like hydrops, congenital heart disease and neural tube defects), birth weight of <1000 g, those who have incomplete documentation (has no appropriate data that measure both maternal and early neonatal parameter) and mothers who are seriously ill and unable to respond to the question were excluded.

### Sample size and sampling procedure

The single population proportion formula was used to determine the sample size with the following assumptions: Where; **n** = Sample size, **Z** = 95% confidence level (Z α/2 = 1.96), **α** = Level of significance 5% (α = 0.05) and **d** = Margin of error 5% (d = 0.05). The prevalence of birth trauma was (P) = 8.1% taken from the previous study done in Jimma University Specialized Hospital, South Western Ethiopia [18], and sample size was 125. The prevalence of birth asphyxia was (P) = 32.9% taken from the previous study conducted in Jimma zone public Hospitals, South West Ethiopia [19] after comparing with other studies done in Ethiopia [8, 20–22], After considering 10% non-response rate, the total sample size was **373.** Finally, from the calculated sample size for the first and second dependent variables, the largest sample size was **373.**

Simple random sampling technique was used to select four hospitals to be included in this study from 11 public hospitals. The number of study unit to be sampled from each selected hospital were determined by proportional to size allocation formula, based on three months report of delivery in each selected hospital. The study subject were selected from list of delivery registration book by using systematic random sampling technique every “K” value = 20, which was obtained through dividing the total number of delivery in three month report from selected hospital to the required sample size. Mothers that delivered more than one baby like twin, one of these babies was selected by using simple random sampling.

### Variables

The dependent variables of the study were birth injuries categorized as birth asphyxia and birth trauma. Whereas, the independent variables were socio demographic variables (maternal age in years, maternal weight, maternal height, pre-pregnancy body mass index (BMI), level of education, place of residence and marital status), medical and obstetrics variables (antenatal care (ANC) follow up, pregnancy type, parity, chronic diabetic mellitus, gestational diabetes mellitus(GDM), chronic hypertension, pregnancy induced hypertension and abruption placenta), intrapartum variables (fetal presentation, duration of labor, cephalopelvic disproportion, intra-partal fetal distress, mode of delivery, cord prolapse, tight nuchal cord induction of labor, meconium stained amniotic fluids, premature rupture of membrane prolonged rupture of membrane, time of birth and qualification of birth attendant) and early neonatal variables (sex, birth weight, head circumferences, APGAR score, need of resuscitation and gestational age).

### Operational definitions

**Birth injury:** Injury to newborns that occur during labor and delivery who has diagnosis of birth trauma, birth asphyxia or both.

**Birth trauma:** Any physical injury to newborns during the entire birth process that can be recognized by clinical physical examination.

**Birth Asphyxia:** Failure to initiate, sustain breathing and not crying at birth and diagnosed based on Apgar score <7 at 5^th^ minutes.

**Fetal distress:** When the fetal heart rate is either <100 or >180 beat/minutes or if there is non-reassurance fetal heart rate pattern.

**Major congenital anomalies:** Are structural or functional abnormalities which are significance effect to reduce life expectancy of newborns such as hydrops, congenital heart disease and neural tube defects.

**ANC follow up:** A programmed clinical visits of a mother at least one during her pregnancy in this study.

**Prolonged labor:** Defined as when the combined duration of the first and the second stage of labor are more than 12 hours in primipara or 8 hours in multipara mothers.

**Premature rupture of membrane:** Rupture of membrane of the amniotic sac and chorion occurred before onset of labor.

**Prolonged rupture of membrane:** Duration of rupture of membrane of the amniotic sac and chorion >18 hours till delivery.

### Data collection tools and procedures

Data collection tools were developed by reviewing different related literatures [8, 10, 18, 20, 22, 23]. Data was collected by Nurses and Midwives at delivery and post-natal ward by using structured interviewer administered questionnaire and checklist. The questionnaire was used to assess socio demographic characteristics of the mothers and medical and obstetrics variables of the mothers. The checklist was used to assess data on intra-partum and early neonatal variables. Birth injuries diagnosis obtained from mothers medical record which was diagnosed by gynecologist/obstetricians and residents. APGAR score was evaluated by resident and Gynacologist.

### Data processing and analysis

After completing data collection, data were categorized, coded, cleaned and recorded. The data was entered by using Epi data version 4.0.2 and exported in to SPSS software version 25. Descriptive statistical analysis such as frequencies, percentages, crosses tabulation and mean were performed. To assess the factors independently associated with birth injury, two regression models (considering the dependent variables to be (i) birth asphyxia and (ii) birth trauma) were used.

Bivariate logistic regression analysis was used to check the association between each independent variable with dependent variable. Then those variables with p-value ≤ 0.25 were entered a multivariable logistic regression model analysis in order to control the confounding factors. To check the correlation between independent variables, multi-colinearity (colinearity diagnostic taste) was done by using the value of variance inflation factors and tolerance. Hosmer and Lemeshow goodness of fit test and omnibus tests of model coefficients were done to test the fitness of the logistic regression in the final model, then it was found good (statistically insignificant value, *P* value >0.05). The strength of association between dependent and independent variables was expressed by using adjusted odds ratio with 95% confidence interval. P-value <0.05 was considered as statistically significance. Finally, the findings were presented by using text, tables and graph.

### Ethical approval and informed consent

The research was reviewed and approved by School of Nursing and Midwifery, Addis Ababa University, College of Health Science, Institutional Review Board (IRB(Protocol number:52/21/SNM)). Permission was also sought from each hospital. Study participants were asked for their willingness to participate in the study after explaining the purpose of the study. Then written informed consent was obtained from each participant. The privacy and confidentiality of information was strictly maintained by not writing the name of study participants on data collection tool.

## Results

### Socio demographic characteristics of the mothers

All of the 373 mothers were give an informed consent to participate with a response rate of 100%. The mean maternal age was 27.28 ± 5.16 SD years of whom 141 (37.8%) of mothers belonged to age groups of 25–29 years. The mean of BMI and height of the mothers were 22.65 ± 3.34SD kg/m^2^ and 156.8 ± 8.5 SD cm respectively (Table 1).

**Table 1:** Socio-demographic characteristics of mothers.

Variables	Category	Frequency (n)	Percentage (%)
**Age group of the mothers**	15–19	22	5.9
	20–24	88	23.6
	25–29	141	37.8
	30–34	73	19.6
	≥35	49	13.1
**Educational status**	No formal education	51	13.7
	Primary education	133	35.7
	Secondary education	108	29.0
	More than secondary	81	21.6
**Residency**	Urban	358	96
	Rural	15	4
**Marital status**	Married	339	90.9
	Divorced	18	4.8
	Single	16	4.3
**Height of the mother (in cm)**	<145	51	13.7
	≥145	322	86.3
**BMI of the mothers (Kg/m^2^)**	<18.5 (underweight)	30	8
	18.5–24.9 (Normal)	264	70.8
	25–29.9 (overweight)	68	18.2
	≥30 (obese)	11	2.9

**Key**: BMI: Body Mass Index

### Medical and obstetric characteristics of the mothers

Among 373 study subjects, 367 (98.4%) of mothers attended ANC follow up during their pregnancy period. Majority of the participants, 312 (83.6%) had four and above ANC follow up. Half 186 (49.9%) of the mothers were primipara. Regarding the chronic medical illness of the mothers, majority of the participants 364 (97.6%) and 369 (98.9%) did not have chronic DM and hypertension respectively. Pregnancy induced hypertension 52 (14%) and gestational diabetes mellitus 40 (10.7%) were the most common obstetrics complication during pregnancy. Around one-tenth 39 (10.5%) of the participants who had pregnancy induced hypertension developed pre-eclampsia. Majority of the mothers 341 (91.4%) had single type of pregnancy and only 32 (8.6%) of the mothers had twin types of pregnancy (Table 2).

**Table 2:** Medical and obstetrics characteristics of the mother.

Variables	Category	Frequency (n)	Percentage (%)
**ANC follow up**	Yes	367	98.4
	No	6	1.6
**Number of ANC follow up**	1–3	55	14.7
	≥4	312	83.6
**Facilities of ANC follow up**	Health centers	262	70.2
	Government hospitals	78	20.9
	Private hospitals	19	5.1
	Private clinic	6	1.6
	NGO clinic	2	0.5
**Parity**	Primipara	186	49.9
	Multipara	187	50.1
**Gravidity**	Primigravida	160	42.9
	Multigravida	213	57.1
**Types of pregnancy**	Single	341	91.4
	Twins	32	8.6
**Medical illness of the mothers**			
**Chronic DM**	Yes	9	2.4
	No	364	97.6
**Chronic hypertension**	Yes	4	1.1
	No	369	98.9
**HIV test done**	Yes	373	100
	No	0	0
**HIV Status**	Positive	8	2.1
	Negative	365	97.9
**Others** *		12	3.21
**Obstetric complication of the mothers**			
**Gestational DM**	Yes	40	10.7
	No	333	89.3
**Pregnancy induced hypertension**	Yes	52	14
	No	321	86
**Types of pregnancy induced hypertension**	Pre-eclampsia	39	10.5
	Eclampsia	13	3.5
**Abruptio placenta**	Yes	8	2.1
	No	365	97.9
**Others** **		26	7

**Key**

* = Anemia, congestive heart failure, thrombocytopenia, asthma and hydronephrosis

** = Oligohydramnious and chorioamnionitis

ANC: Antenatal Care, DM: Diabetes Mellitus, HIV: Human Immunodeficiency virus, NGO: Non-governmental organization.

### Intrapartum related factors

According to the result of this study, majority 342 (91.7%) of the newborns were at vertex presentation. Around 88 (23.6%) of the newborns had intrapartum fetal distress. Among the total participated mothers, above two third 254 (68.1%) and 60 (16.1%) had spontaneous and induced onset of labor respectively. In addition to this, about 59 (15.8%) of the mothers did not experience any onset of labor during delivery i.e. delivered by elective cesarean section.

Nearly one third 119 (31.9%) of the mothers had prolonged duration of labor. Furthermore, one quarters 90 (24.1%), 54 (14.5%) and 81(21.7%) of the mothers faced premature rupture of membranes, prolonged rupture of membranes (≥18 hours) and meconium stained amniotic fluid respectively. More than half 217(58.2%) and 37(9.9%) of the newborns were delivered by cesarean section and instrumental delivery respectively. Regarding to cord problem, only 8 (2.1%) and 13 (3.5%) of the newborns developed cord prolapse and tight nuchal cord during delivery respectively. Majority of the delivery 184 (49.3%) and 135 (36.2%) attended by residents and midwifes respectively (Table 3).

**Table 3:** Intra-partum factors of mother for the study of prevalence of birth injuries and associated factors.

Variables	Category	Frequency	Percentages (%)
**Fetal presentation**	Vertex presentation	342	91.7
	Breech presentation	23	6.2
	Face presentation	5	1.3
	Brow presentation	3	0.8
**Intrapartal fetal distress**	Yes	88	23.6
	No	285	76.4
**CPD**	Yes	9	2.4
	No	364	97.6
**Condition of labor**	Spontaneous	254	68.1
	Induced	60	16.1
	No labor (elective c/s)	59	15.8
**Duration of labor**	Normal	195	52.3
	Prolonged	119	31.9
	No labor	59	15.8
**Premature rupture of membrane**	Yes	90	24.1
	No	283	75.9
**Duration of rupture of membrane**	<18 hours	317	85
	≥ 18 hours	56	15
**Color of amniotic fluid**	Clear	292	78.3
	Meconium stained	81	21.7
**Mode of delivery**	SVD	119	31.9
	Instrumental delivery	37	9.9
	C/S	217	58.2
**Cord prolapse**	Yes	8	2.1
	No	365	97.9
**Tight nuchal cord**	Yes	13	3.5
	No	360	96.5
**Qualifications of birth attendant**	Gynecologists/obstetricians	54	14.5
	Residents	184	49.3
	Midwifes	135	36.2
**Time of birth**	Day time birth	230	61.7
	Night time birth	143	38.3

**Key**: CPD: Cephalopelvic Disproportion, C/S: Caesarian Section, SVD: Spontaneous Vaginal Delivery

### Early neonatal related factors

Of the total newborn babies, 225 (60.3%) of them were males. More than three quarters 288 (77.2%) of the newborn babies’ gestational age was in the range of 37–42 weeks at birth. The mean gestational age at the time of birth was 39.45 ± 2.52 SD weeks. Besides, majority 285 (76.4%) of the participants had normal birth weight (2500–3999) gram and the average birth weight of the newborn babies was 3119.09 ± 649.25 SD grams. 336 (90.1%) of the participants had normal head circumference (33–38 cm) respectively. Moreover, around 52 (13.9%) of the newborns were unable to cry immediately after birth. About 321 (86.1%) of the newborn babies had normal Apgar score at fifth minutes after birth (7–10). Additionally, 43(11.5%) and 9 (2.4%) of the participants had moderate (4–6) and low (0–3) APGAR score respectively. Out of the study population,52 (13.9%) of the newborns needed resuscitation after birth (Table 4).

**Table 4:** Early neonatal related factors of newborns delivered in public hospitals, Addis Ababa, Ethiopia, 2021 (n = 373).

Variables	Category	Frequency	Percentages (%)
**Sex**	Male	225	60.3
	Female	148	39.7
**Gestational age**	<37 weeks (preterm)	44	11.8
	37–42 weeks (term)	288	77.2
	>42 weeks (post term)	41	11
**Birth weight**	<2500 gram	54	14.5
	2500–3999 gram	285	76.4
	≥4000 gram	34	9.1
**Head circumference**	<33 cm	21	5.6
	33–38 cm	336	90.1
	>38 cm	16	4.3
**Cry after birth**	Yes	321	86.1
	No	52	13.9
**APGAR score (1**^**st**^ **minutes)**	0–3 (low)	17	4.6
	4–6 (moderate)	62	16.6
	7–10 (normal)	294	78.8
**APGAR score (5**^**th**^ **minutes)**	0–3	9	2.4
	4–6	43	11.5
	7–10	321	86.1
**Resuscitation after birth**	Yes	52	13.9
	No	321	86.1

### Prevalence of birth injuries

**Fig 1:**
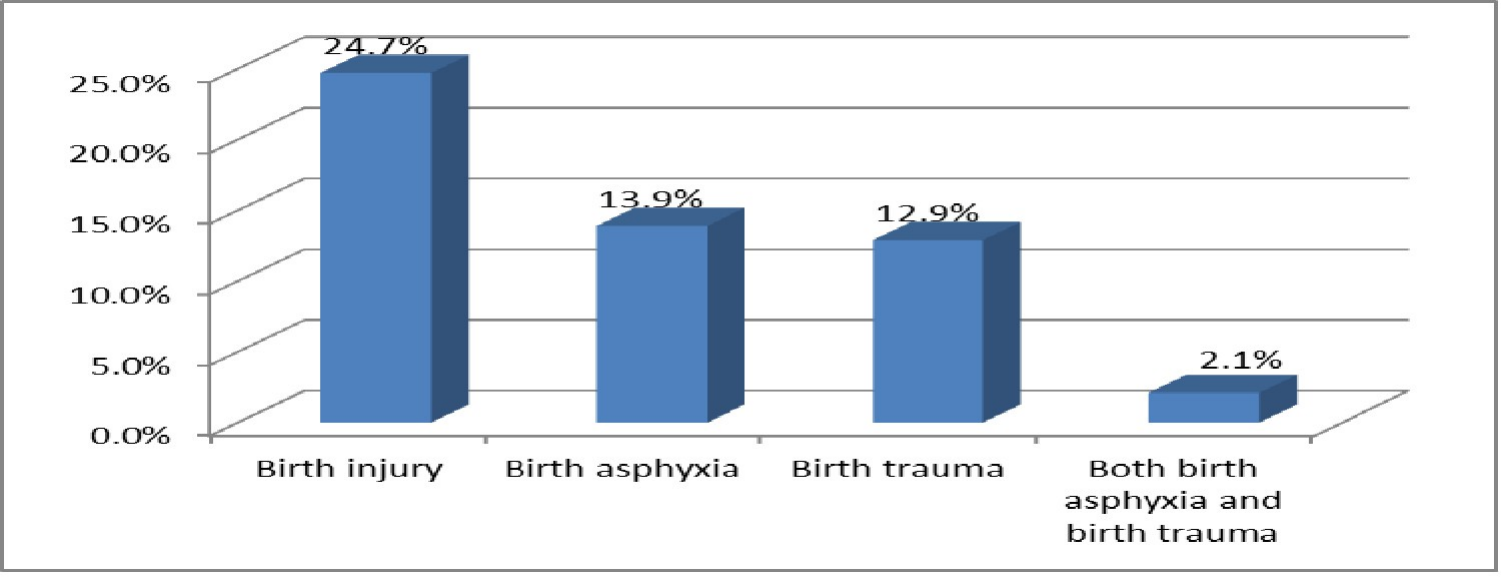
Prevalence of birth injury among newborns delivered in public hospitals Addis Ababa, Ethiopia, 2021.

The overall prevalence of birth injury was found to be 92 (24.7%) of the total study participants in this study. Birth asphyxia and birth trauma were identified in 52 (13.9%) and 48 (12.9%) of these babies, respectively. A total of eight newborns (2.1%) suffered from both birth asphyxia and birth trauma (Fig 1).

Among those newborns who diagnosed with birth trauma, the most common types were extra cranial trauma 39 (81.2%), neurological trauma 13 (27%) and soft tissue trauma 10 (21%). From extra cranial trauma, more than half, 20 (51.2%) and 10 (25.6%) of the newborns babies developed subgalial hemorrhage and cephalhaematoma respectively. Among neurological trauma and soft tissue trauma, the largest proportions contributed by facial palsy 8 (61.5%) and facial &skin bruises 5(50%) respectively. Furthermore, 14 (29.2%) newborns developed two types of birth trauma (Table 5).

**Table 5:** Common types of birth trauma among newborns delivered in public hospitals, Addis Ababa, Ethiopia, 2021.

Types of birth trauma	Frequency (n)	Percentages (%) from newborn with birth trauma (n = 48)	Percentages (%) from study population (n = 373)
**Extra cranial trauma**			
Caput succedaneum	9	18.8	2.41
Cephalhaematoma	10	20.8	2.68
Subgalial hemorrhage	20	41.7	5.36
**Neurologic trauma**			
Erb’s palsy	5	10.4	1.3
Facial palsy	8	16.7	2.1
**Soft tissue trauma**			
Facial and skin bruises	5	10.4	1.3
Skin laceration	3	6.3	0.8
Sub-conjuctival hemorrhage	2	4.2	0.5

### The associated factors of birth asphyxia

In multivariable logistic regression analysis, short height of the mothers, intrapartal fetal distress, cord prolapse, tight nuchal cord, birth attended by residents, male sex and low birth weight of the newborns were the most contributing factors of birth asphyxia (Table 6).

**Table 6:** Bivariate and multivariable logistic regression analysis for the associated factors of birth asphyxia.

Variables	Category	Birth asphyxia		COR(95% CI)	AOR (95% CI)
		Yes(n = 52)	No(n = 321)		
**Age groups of mothers**	15–19	4(7.7%)	18(5.6%)	1	1
	20–24	14(26.9%)	74(23.1%)	0.85(0.25–0.28)	0.71(0.11–4.46)
	25–29	19(36.5)	122(38%)	0.70(0.21–2.29)	0.67(0.10–4.30)
	30–34	4(7.7%)	69(21.5%)	**0.26(0.05–1.14)** *	0.59(0.06–5.48)
	≥35	11(21.2%)	38(11.8%)	1.30(0.36–4.65)	3.85(0.44–33.0)
**Educational status of mothers**	No formal education	9(17.3%)	42(13.1%)	**3.25(1.02–10.3)** *	1.09(0.22–5.41)
	Primary	21(40.4%)	112(34.9%)	**2.85(1.03–7.88)** *	1.38(0.37–5.03)
	Secondary	17(32.7%)	91(28.3%)	**2.84(1.00–8.05)** *	1.33(0.32–5.51)
	Above secondary	5(9.6%)	76(23.7%)	1	1
**BMI (Kg/m^2^)**	<18.5	4(7.7%)	26(8.1)	1	1
	18.5–24.9	26(50%)	238(74.1%)	0.71(0.23–2.19)	1.06(0.18–6.06)
	25–29.9	19(36.5%)	49(15.3%)	**2.52(0.77–8.18)** *	2.08(0.31–13.5)
	≥30	3(5.8%)	8(2.5%)	2.43(0.44–13.2)	3.06(0.29–32.4)
**Height of the mothers**	<145 cm	22(42.3%)	29(9%)	**7.38(3.78–14.4)** *	**10.7(3.59–32.4)** **
	≥145 cm	30(57.7%)	292(91%)	1	1
**Parity**	Primipara	31(59.6%)	155(48.3%)	**1.58(0.87–2.86)** *	2.04(0.72–5.77)
	Multipara	21(40.4%)	166(51.7%)	1	1
**GDM**	Yes	10(19.2%)	30(9.3%)	**2.31(1.05–5.06)** *	2.24(0.52–9.67)
	No	42(80.8%)	291(90.7%)	1	1
**Types of pregnancy**	Single	50(96.2%)	291(90.7%)	**2.57(0.59–11.1)** *	4.48(0.49–40.7)
	Twine	2(3.8%)	30(9.3%)	1	1
**Abruptio placenta**	Yes	4(7.7%)	4(1.2%)	**6.6(1.59–27.2)** *	5.30(0.52–54.0)
	No	48(92.3%)	317(98.8%)	1	1
**Intrapartal fetal distress**	Yes	26(50%)	62(19.3%)	**4.17(2.26–7.68)** *	**4.74(1.81–12.4)** **
	No	26(50%)	259(80.7%)	1	1
**CPD**	Yes	4(7.7%)	5(1.6%)	**5.26(1.36–20.3)** *	5.08(0.85–30.3)
	No	48(92.3%)	316(98.4%)	1	1
**Condition of labor**	Spontaneous	42(80.8%)	212(66%)	**3.78(1.13–12.6)** *	6.73(0.88–51.2)
	Induced	7(13.5%)	53(16.5%)	**2.5(0.61–10.2)** *	2.88(0.30–27.4)
	No labor	3(5.8%)	56(17.4%)	1	1
**Duration of labor**	Normal	24(49%)	171(64.5%)	1	1
	Prolonged	25(51%)	94(35.5%)	**1.89(1.02–3.5)** *	1.80(0.70–4.62)
	No labor	3(5.8%)	56(17.4%)	**0.38(0.11–1.31)** *	0.54(0.35–2.42)
**Duration of rupture of membranes**	<18 hours	41(78.8%)	276(86%)	1	1
	≥18 hours	11(21.2%)	45(14%)	**1.64(0.78–3.43)** *	1.05(0.33–3.32)
**Color of amniotic fluids**	Clear	33(63.5%)	259(80.7%)	1	1
	Meconium stained	19(36.5)	62(19.3%)	**2.4(1.28–4.51)** *	1.95(0.72–5.27)
**Cord prolapse**	Yes	4(7.7%)	4(1.2%)	**6.6(1.59–27.2)** *	**7.7 (1.45–34.0)** **
	No	48(92.3%)	317(98.8)	1	1
**Tight nuchal cord**	Yes	7(13.5%)	6(1.9%)	**8.16(2.62–25.3)** *	**9.2 (4.9–35.3)** **
	No	45(86.5%)	315(98.1%)	1	**1**
**Qualification of birth attendant**	Gynecologist	14(26.9%)	40(12.5%)	1	1
	Residents	25(48.1%)	159(49.5%)	**0.44(0.21–0.94)** *	**0.19(0.05–0.68)** **
	Midwifes	13(25%)	122(38%)	**0.3(0.13–0.7)** *	0.62(0.15–2.56)
**Time of birth**	Day time	20(38.5%)	210(65.4%)	1	1
	Night time	32(61.5%)	111(34.6%)	**3.02(1.65–5.53)** *	1.81(0.73–4.51)
**Sex**	Male	39(75%)	186(57.9%)	**2.17(1.11–4.23)** *	**3.84(1.30–11.3)** **
	Female	13(25%)	135(42.1%)	1	1
**Birth weight**	<2500 g	12(23.1%)	42(13.1%)	**2.10(1.01–4.39)** *	**5.28(1.58–17.6)** **
	2500–3999 g	34(65.4%)	251(78.2%)	1	1
	≥4000g	6(11.5%)	28(8.7%)	1.58(0.61–4.09)	0.29(0.04–1.75)

Hosmer and Lemeshow test, P-value = 0.758.

*statistically significant by COR at P-value ≤0.25.

** Statistically significant by AOR at P-value<0.05.

**Key**: BMI: Body Mass Index, CPD: Cephalopelvic Disproportion, GDM: Gestational Diabetes Mellitus, COR: Crude Odds Ratio, AOR: Adjusted Odds Ratio

The occurrence of birth asphyxia was 10.7 times (AOR = 10.7, 95% CI: 3.59–32.4) higher to occur among neonates born from mothers with short height (<145 cm) in relative to neonates born from mothers with height >145 cm. Similarly, the odds of birth asphyxia among mothers who had intrapartal fetal distress were nearly five times (AOR = 4.74, 95% CI: 1.81–12.4) higher than their counterpart. Furthermore, newborns who had cord prolapse and nuchal cord during delivery were 7.7 times (AOR = 7.7, 95% CI: 1.45–34.0) and 9.2 times (AOR = 9.2, 95% CI: (4.9–35.3) more likely experienced birth asphyxia compared to those neonates born without cord prolapse and nuchal cord respectively.

Labor attended by residents were 81% less likely (AOR = 0.19, 95% CI: 0.05–0.68) to encounter birth asphyxia among newborns compared to those labor attended by gynecologist/obstetricians. Besides, the odds of experiencing birth asphyxia was nearly four times higher (AOR = 3.84, 95% CI: 1.30–11.3) among male newborns comparing to female newborns. In addition to this, low birth weight newborns were 5.28 more likely (AOR = 5.28, 95% CI: 1.58–17.6) to develop birth asphyxia relative to normal birth weight newborns (Table 6).

### The associated factors of birth trauma

To control the effect of confounding, multivariate analysis were done and factors independently associated with birth trauma were GDM, prolonged duration of labor, instrumental delivery and night time birth (Table 7).

**Table 7:** Bivariate and multivariable logistic regression analysis for the associated factors of birth trauma.

Variables	Category	Birth trauma		COR (95% CI)	AOR (95% CI)
		Yes (n = 48)	No (n = 325)		
**BMI (Kg/m^2^)**	<18.5	3(6.3%)	27(8.3%)	1	1
	18.5–24.9	24(50%)	240(73.8%)	0.90(0.25–3.18)	1.55(0.21–11.2)
	25–29.9	18(37.5%)	50(15.4%)	**3.24(0.87–11.9)** *	1.59(0.17–14.5)
	≥30	3(6.3%)	8(2.5%)	**3.37(0.56–20.0)** *	3.09(0.23–41.5)
**Height of the mothers**	<145 cm	13(27.1%)	38(11.7%)	**2.8(1.36–5.76)** *	1.73(0.54–5.55)
	≥145 cm	35(72.9%)	287(88.3%)	1	1
**Number of ANC follow up**	1–3	10(20.8%)	45(13.8%)	1	1
	≥4	36(75%)	276(84.9%)	**0.58(0.27–1.26)** *	0.37(0.13–1.10)
**GDM**	Yes	16(33.3%)	24(7.4%)	**6.27(3.02–13.0)** *	**5.01(1.38–18.1)** **
	No	32(66.7%)	301(92.6%)	1	1
**Fetal presentation**	Vertex	41(85.4%)	301(92.6%)	**0.06(0.006–0.76)** *	0.04(0.002–1.08)
	Breech	1(2.1%)	22(6.8%)	**0.02(0.001–0.51)** *	0.11(0.002–5.55)
	Face	4(8.3%)	1(0.3%)	2.00(0.07–51.5)	3.36(0.05–21.7)
	Brow	2(4.2%)	1(0.3%)	1	1
**Duration of labor**	Normal	19(36.6%)	176(54.2%)	1	1
	Prolonged	29(60.4%)	90(27.7%)	**2.98(1.58–5.61)** *	**3.74(1.52–9.20)** **
	No labor	0(0%)	59(18.2%)		
**Mode of delivery**	SVD	13(27.1%)	106(32.6%)	1.44(0.65–3.08)	1.15(0.39–3.32)
	Instrumental	18(37.5%)	19(5.8%)	**11.1(4.94–25.1)** *	**10.6(3.45–32.7)** **
	C/S	17(35.4%)	200(61.5%)	1	1
**Time of birth**	Day time	13(27.1%)	217(66.8%)	1	1
	Night time	35(72.9%)	108(33.2%)	**5.41(2.74–10.6)** *	**4.82(1.84–12.6)** **
**Sex**	Male	34(70.8%)	191(58.8%)	**1.7(0.88–3.29)** *	0.99(0.39–2.51)
	Female	14(29.2%)	134(41.2%)	1	1
**Birth weight**	<2500 g	4(8.5%)	50(15.4%)	0.70(0.23–2.09)	0.36(0.06–2.21)
	2500–3999 g	29(60.4%)	256(78.8%)	1	1
	≥4000 g	15(31.3%)	19(5.8%)	**6.96(3.20–15.1)** *	1.70(0.41–7.00)
**Head circumference**	<33 cm	3(6.3%)	18(5.5%)	1	1
	33–38 cm	36(75%)	300(92.3%)	0.72(0.20–2.56)	0.12(0.01–1.09)
	>38 cm	9(18.8%)	7(2.2%)	**7.71(1.60–37.1)** *	1.25(0.09–17.1)

Hosmer and Lemeshow test, P-value = 0.85.

* = Statistically significant by COR at P-value ≤0.25.

** = Statistically significant by AOR at P-value<0.05.

**Key:** ANC: Antenatal Care, BMI: Body Mass Index, C/S: Caesarian Section, GDM: Gestational Diabetes Mellitus, COR: Crude Odds Ratio, AOR: Adjusted Odds Ratio

The odds of birth trauma were 5 times (AOR = 5.01, 95% CI: 1.38–18.31) higher among neonates born from mothers with gestational diabetic mellitus compared to those born from mothers who did not experience gestational diabetic mellitus. Regarding duration of labor, neonates born from mothers who had prolonged labor were 3.74 times (AOR = 3.74, 95% CI: 1.52–9.20) more likely to develop birth trauma when compared to those born from mother with normal duration of labor. Those neonates born via instrumental assisted were nearly 10.6 times (AOR = 10.6, 95% CI: 3.45–32.7) more susceptible to experience birth trauma than neonates delivered via caesarian section. Moreover, neonates delivered during the night time were nearly five times (AOR = 4.82, 95% CI: 1.84–12.6) more likelihood of acquiring birth trauma than neonates born during the day time (Table 7).

## Discussion

Birth injury is the primary cause of morbidity among live birth newborns in our study area. The prevalence of birth injury differs widely from place to place. In this study the burdens and associated factors of birth injury among live birth newborns at Addis Ababa Public Hospital are reported. Short height of the mothers, intrapartal fetal distress, cord prolapse, tight nuchal cord, birth attended by residents, male sex and low birth weight where found to be a significant predictors of birth asphyxia. Whereas, birth trauma was significantly associated with gestational diabetic mellitus, prolonged duration of labor, instrumental delivery and night time birth.

### Prevalence of birth injury

The overall prevalence of birth injury among live birth newborns was 24.7% with 95% CI (20.1–29.0). It was higher than studies conducted in Indian, Iran, Nigeria and Jimma (11.76%, 2.2%, 5.7%, 15.4% respectively) [10, 12, 13, 18]. This variation might be due to difference in sample size and study area (this study conducted in referral hospitals where more complicated cases and referred from different setting that could increase the prevalence of birth injury in the study area).

In this study the prevalence of birth asphyxia was 13.9% with 95% CI (10.5–17.7). This finding was higher compared to studies conducted in Jimma 8.1% [18], Dire Dawa 2.5% [20] and South Indian 5.29% [13]. However, it was lower than the studies conducted in Jimma zone public hospitals 32.9% [19], Debre Tabor 28.35% [22], North East Amhara 22.6% [21] and Hossana 15.1% [8]. Similarly, this finding also lower as compared to the studies conducted in Iran 16.8% [10] and Nigeria 39.3% [12]. The possible reason might be difference in sample size, using different definition of birth asphyxia (some studies used 1^st^ minutes APGAR score, but this study used 5^th^ minutes APGAR score to define birth asphyxia), variation of the study area and variation in distribution of skilled birth attendant in different setting.

The current study showed that the prevalence of birth trauma was 12.9% with 95% CI (9.7–16.4). This finding was higher as compared to the studies done in USA 2.9% [15], Pakistan 4.11% [24], India 1.54% [16] and Jimma 8.1% [18], However, this result was lower than studies conducted in Nigeria 67.2% [25]. This might be due to difference in study design, sample size, study population and variation in diagnosis of birth trauma, i.e. this study used birth trauma that was diagnosed only by physical examination but other studies included birth trauma diagnosed by both physical examination and radiological.

The most common birth trauma seen in the current study was extra cranial trauma 39 (81.2%), neurological trauma 13 (27%) and soft tissue trauma10 (21%). Subgalial hemorrhage 41.7% and cephalhaematoma 20.8% were the most common birth trauma. This finding was higher than studies done in Jimma and Nigeria, they were found that the most prevailing birth trauma was subgalial hemorrhage which accounts 20% and 13.1% respectively. The possible reason might be in the current study, instrumental delivery is significantly associated with birth trauma but not in study conducted in Jimma [18]. In addition to this, there was low rate of instrumental assisted delivery due to fear of cultural belief, so most women prefer to deliver by spontaneous vaginal delivery in study conducted in Nigeria [12].

Cephalhaematoma was the second common types of birth trauma diagnosed in around 20.8% of the newborns, it was lower when compared to studies done in Iran [10] and India [26], they were found that the most common type of birth trauma was cephalhaematoma accounts 57.2% and 38.7% respectively. However this finding was higher than study done in Nigeria 16.4% [12] and Pakistan 2.14% [24]. This might be due to differs in the skill of birth attendant and frequency of instrumental delivery.

In this study, facial palsy was the most prevailing among neurological trauma. This finding was supported by studies carried out in Iran [10], Indian [16], Bombay Hospital [26] and Nigeria (Maiduguri) [12]. The possible reason may be the fact that facial palsy occur during difficult delivery when forceps are applied and leads to paralysis of seventh cranial nerve.

### The associated factors of birth asphyxia

Factors independently associated with birth asphyxia were short height of the mothers, intrapartal fetal distress, cord prolapse, tight nuchal cord, birth attended by residents, male sex of the newborns and low birth weight of the newborns.

The occurrence of birth asphyxia was 10.7 times (AOR = 10.7, 95% CI: 3.59–32.4) higher among neonates born from mothers with short height (<145 cm) in relative to neonates born from mothers with height >145 cm. This finding was supported by studies conducted in Swedish [27], Uganda [28] and Ethiopia [29]. This could be due to the fact that those mothers who had short height may have short stature that impair the progress of descent of the fetal head and leads to prolong the duration of labor. This predisposes the newborn for birth asphyxia.

Our study also identified that intrapartal fetal distress was significantly associated with birth asphyxia. The odds of birth asphyxia among mothers who had intrapartal fetal distress were nearly five times (AOR = 4.74, 95% CI: 1.81–12.4) higher as compared to those mothers without history of intrapartal fetal distress. This finding was almost similar to the previous studies conducted in Gonder [30] and Addis Ababa [24]. The likely reason is either fetal tachycardia or fetal bradycardia is the main cause for fetal-placental oxygen deprivation that exposes the newborn for birth asphyxia. Usually it’s an indication for emergency cesarean section. But this finding is lower than the study conducted in Jimma, Ethiopia neonates with intrapartal fetal distress had 6.4 times more likely to develop birth asphyxia when compare to neonates without intrapartal fetal distress [18]. This difference may be due to variation in study setting and quality of the obstetric care.

The occurrence of birth asphyxia was also independently associated with cord prolapse and tight nuchal cord. Newborns who had cord prolapse during delivery were 7.7 times (AOR = 7.7, 95% CI: 1.45–34.0) and tight nuchal cord during delivery were 9.2 times (AOR = 9.2, 95% CI: 4.9–35.3) more likely experienced birth asphyxia compared to their counterpart. This finding was supported with the previous studies conducted in USA [31], Hossana [8] and Jimma [19]. This could be due to the fact that compression of the cord may impair blood flow to the fetus and compromise the fetal oxygenation; as a result the chance of occurrence of birth asphyxia will be more likely.

Labor attended by residents were 81% less likely (AOR = 0.19, 95% CI: 0.05–0.68) to encounter birth asphyxia among newborns compared to those labor attended by gynecologist/obstetricians. This might be due to since the study was conducted in teaching hospitals; most labor was attended by residents, but labor attended by gynecologists/obstetricians was critical cases/ consulted case that was unable to handle by residents. In addition to the above reason, there may be variation in skill of neonatal resuscitation b/n resident and gynacologist, that determine the newborns outcome [32]. This finding was inconsistent with study conducted in Debre Tabor, Ethiopia neonates delivered by Midwives 56.2% developed birth asphyxia [24]. The difference may be due to variation in study setting and distribution of skilled birth attendant i.e. trained in neonatal resuscitation.

The odds of experiencing birth asphyxia was nearly four times higher (AOR = 3.84, 95% CI: 1.30–11.3) among male newborns comparing to female newborns. This finding was supported by study conducted in Washington, American [33] and Ayder Hospital, Ethiopia [34]. This might be due to biological difference makes male more at risk for birth asphyxia and it needs further investigation. In addition to this, low birth weight newborns were 5.28 more likely (AOR = 5.28, 95% CI: 1.58–17.6) to develop birth asphyxia relative to those who had normal birth weight. It was in agreement with study conducted in Addis Ababa [35], Gonder [30] and Jimma [19]. This might be clarified by the fact that most low birth weight neonates delivered during preterm gestation that might have immature lung and unable to pass the transition period without difficulty of breathing.

### The associated factors of birth trauma

The other dependent variable is birth trauma and the associated factors were found to be GDM, prolonged duration of labor, instrumental delivery and night time birth. The odds of birth trauma were 5 times (AOR = 5.01, 95% CI: 1.38–18.1) higher among neonates born from mothers with gestational diabetic mellitus compared to those born from mothers who did not experience gestational diabetic mellitus. This finding was consistent with the studies conducted in Nigeria [25] and Turkey [36]. This might be due to the truth that, one of the complications of infant of diabetic mothers is macrosomia, and this will predispose the newborn for mechanical birth trauma that is why it’s the main reason for emergency C/s.

Neonates born from mothers who had prolonged labor were 3.74 times (AOR = 3.74, 95% CI: 1.52–9.20) more likely to develop birth trauma when compared to those born from mother with normal duration of labor. This finding was supported by studies done in Nigeria [25], Indian [16] and Bombay hospital [26]. This is due to the fact that when there is prolonged labor, the women may experience tiredness and unable to progress the labor. Therefore, to prevent fetal distress, the birth attendant may apply forceps or vacuum to assist the labor. All these difficulty may leads to birth trauma.

Another contributing factor significantly associated with birth trauma was instrumental delivery. Those neonates born via instrumental assisted were 10.6 times (AOR = 10.6, 95% CI: 3.45–32.7) more susceptible to experience birth trauma than neonates delivered via cesarean section. This finding was in agreement with studies conducted in Bombay Hospital [26], Indian [13] and Nigeria [12]. The likely reason was due to the fact that, application of forceps and vacuum on the fetal head may expose to extra cranial hemorrhage, intra cranial hemorrhage and soft tissue abrasion/laceration. All these complication may leads to birth trauma. But, this finding was higher than study done in Pakistan [24], neonates delivered by instrument assisted were 2.14 times (AOR = 2.14) more likely to develop birth trauma than neonates delivered via cesarean section. This difference might be due to variation in study setting and skill of birth attendant.

Night time delivery was another contributing factor for birth trauma. Neonates delivered during the night time were nearly five times (AOR = 4.82, 95% CI: 1.84–12.6) more likelihood of acquiring birth trauma than neonates born during the day time. This finding was supported by study conducted Indian [16]. This is possibly justified by the number of birth attendant assigned during duty hours were few that makes them unable to accomplish the overburden during night time, expert in the field/gynecologist may not arrived on time for consulted cases and it might be large proportion of referred cases during night time.

## Conclusion and recommendation

The overall prevalence of birth injury in this study was 24.7%, which is still higher than the previous studies conducted in developing countries. Each birth asphyxia and birth trauma constitutes 13.9% and 12.9% respectively. Birth asphyxia was independently associated with short height of the mothers, intrapartal fetal distress, cord prolapse, tight nuchal cord, birth attended by residents, male sex of the newborns and low birth weight of the newborns. In addition to this, birth trauma was independently associated with GDM, prolonged duration of labor, instrumental delivery and night time birth. However, the finding of this study could only be generalized to this cohort womens–newborns in the study setting. The medical service provided to the mothers and newborns during delivery is important to reduce the overall prevalence of birth injury and its burden.

Therefore, most of the above contributing factors are preventable and strong effort must be done to improve prenatal care and the delivery service which are vital to reduce the occurrence of birth injury and its complications.

## Supporting information

S1 Data Raw data of the study. (SAV)

S1 File Data collection tools used to assess prevalence of birth injuries and associated factors among newborns delivered in public hospitals Addis Ababa, Ethiopia, 2021. Crossectional study.
(DOCX)

## List of abbreviations

ANC: Antenatal Care
APGAR: Appearance, Pulse rate, Grimace, Activity and Respiration
BMI: Body Mass Index
CI: Confident Interval
CPD: : Cephalopelvic disproportion
C/S: Caesarian Section
EDHS: Ethiopian Demographic Health Survey
GA: Gestational Age
GDM: Gestational Diabetes Mellitus
GMH: Gandhi Memorial Hospital
ICD: International classification of disease
OR: Odds Ratio
SPHMMC: St. Paul Hospital Millennium Medical College
TASH: Tikur Anbessa Specialized Hospital
WHO: World Health Organization
Y-12HMC: Yekatit 12 Hospital Medical College

## References

[1] Akangire G, Carter B. Birth Injuries in Neonates. Pediatr Rev. 2016;37(11). doi: 10.1542/pir.2015-0125 27803142

[2] Rosenberg AA. Traumatic Birth Injury. Neoreviews. 2020;4(10).

[3] OF Njokanma OK. mechanical birth trauma-An evaluation of predisposing factors at ogun state univerity teaching hospital,sagamu. Niger J Paediatr. 2012;29(3):61–5.

[4] Chaturvedi A, Chaturvedi A, Stanescu AL. Mechanical birth-related trauma to the neonate: An imaging perspective. Insights Imaging. 2018;9:103–18. doi: 10.1007/s13244-017-0586-x 29356945 PMC5825313

[5] Tekes A, Pinto PS. Birth-Related Injury to the Head and Cervical Spine in Neonates. Magn Reson Imaging Clin N Am. 2011;19:777–790. doi: 10.1016/j.mric.2011.08.004 22082737

[6] WHO. Basic newborn resuscitation_ A practical guide,Maternal and Newbornhealth/safe motherhood unit division of reproductive health. WHO, Geneva; 2020.

[7] The American College of Obstetricians and Gynecologists & American Academy of Pediatrics. The Apgar Score. Committee opinion. Am Acad Pediatr Am Coll Obstet Gynecol. 2017;126(644):e52–5.

[8] Abdo RA, Halil HM, Kebede BA, Anshebo AA. Prevalence and contributing factors of birth asphyxia among the neonates delivered at Nigist Eleni Mohammed memorial teaching hospital, Southern Ethiopia: a cross- sectional study. BMC Pregnancy Childbirth. 2019;6:1–7. doi: 10.1186/s12884-019-2696-6 31888542 PMC6937931

[9] Ethiopian Federal Ministery of Health. Neonatal Intensive Care Unit (NICU) Training Participants ‘ Manual. 2014;

[10] Abedzadeh-kalahroudi M, Talebian A, Jahangiri M. Incidence of Neonatal Birth Injuries and Related Factors in Kashan, Iran. Arch Trauma Res. 2015;4(1):e22831. doi: 10.5812/atr.22831 26064868 PMC4460260

[11] WHO. ICD-10 Version_2016.

[12] Ba I, Ag F, Simon P. Incidence and characteristics of neonatal birth injuries in Maiduguri North-Eastern Nigeria. Niger J Paediatr. 2018;45(2):99–105.

[13] u-zama R, Jeergal NA, Thobbi AN, Vijay Katti S. a Clinical Study of Neonatal Birth Injuries in a Tertiary Care Hospital-Nicu, Bijapur. Indian J Child Health. 2020;7(7):288–90.

[14] Liu L, Mathers C, Oza S, Chu Y, Black B, Cousens S, et al. MCEE-WHO methods and data sources for child causes of death 2000–2015. World Heal Organ. 2016;1:20.

[15] Sauber-Schatz EK, Markovic N, Weiss HB, Bodnar LM, Wilson JW, Pearlman MD. Descriptive epidemiology of birth trauma in the United States in 2003. Paediatr Perinat Epidemiol. 2010;24(2):116–24. doi: 10.1111/j.1365-3016.2009.01077.x 20415766

[16] Ray S, Mondal R, Samanta M, Hazra A, Sabui T, Debnath A, et al. Prospective study of neonatal birth trauma: Indian perspective. J Clin Neonatol. 2016;5(2):91.

[17] Darmstadt GL, Hussein MH, Winch PJ, Haws RA, Gipson R, Santosham M. Practices of rural Egyptian birth attendants during the antenatal, intrapartum and early neonatal periods. J Heal Popul Nutr. 2018;26(1):36–45. PMC2740680 18637526

[18] Tesfaye W, Netsanet Workneh EG. Birth injury and associated factors in jimma university specialized hospital, southwest ethiopia. Ethiop J Pediatr Child Health. 2016;xii(1).

[19] Wayessa Z. J., Belachew T., & Joseph J. (2018). Birth asphyxia and associated factors among newborns delivered in Jimma zone public hospitals, Southwest Ethiopia: A cross sectional study. *Journal of Midwifery and Reproductive Health*, 6(5), 2189–1295. 10.22038/JMRH.2018.10483.

[20] Abdurashid N. Prevalence of Birth Asphyxia and Associated Factors among Neonates Delivered in Dilchora Referral Hospital, in Dire Dawa, Eastern Ethiopia Clinics in Mother and Child Health. Clin Mother Child Heal. 2017;14(4).

[21] Woday A, Muluneh A, Denis CS. Birth asphyxia and its associated factors among newborns in public hospital, northeast,Amhara, Ethiopia. PLoS One. 2019;14(12):113. doi: 10.1371/journal.pone.0226891 31860643 PMC6924666

[22] Bayih WA, Yitbarek GY, Aynalem YA, Abate BB. Prevalence and associated factors of birth asphyxia among live births at Debre Tabor General Hospital, North Central Ethiopia. 2020;2:1 12. doi: 10.1186/s12884-020-03348-2 33115413 PMC7594464

[23] Unicef for every child.Maternal and Newborn Health Disparities Ethiopia.2015. Ethiopia.

[24] Shabbir S, Zahid M. Risk factors and incidence of birth trauma in tertiary care hospital of Karachi. Pakistan J Med Heal Sci. 2015;31(1):66–9.

[25] Osinaike BO, Akinseye LOO, Akiyode OR, Anyaebunam C, Kushimo O. Prevalence and predictive factors of birth traumas in neonates presenting to the children emergency center of a tertiary center in Southwest, Nigeria. 2017;167–72.

[26] Warke C, Malik S. Birth Injuries -A Review of Incidence, Perinatal Risk Factors and Outcome. Bombay Hosp J. 2012;54(2).

[27] Liljeström LB asphyxia. F scalp blood sampling and risk factors for hypoxic ischemic encephalopathy. DCS of UD from the F of M 1435. 81 pp. UAUUI 201. Birth asphyxia. 2018.

[28] Munabi IG, Luboga SA, Mirembe F. A cross sectional study evaluating screening using maternal anthropometric measurements for outcomes of childbirth in Ugandan mothers at term. BMC Res Notes. 2015;8(1):1–8. doi: 10.1186/s13104-015-1183-z 26032185 PMC4467626

[29] Gizachew Y. et al.Obstructed labor and its effect on adverse maternal and fetal outcomes in Ethiopia: A systematic review and meta-analysis.2022;Plos one; 14, 1–24. 10.1371/journal.pone.0275400 PMC9524671 36178921

[30] Wosenu L, Worku AG, Teshome DF, Gelagay AA. Determinants of birth asphyxia among live birth newborns in University of Gondar referral hospital, northwest Ethiopia: A case-control study. PLoS One. 2018;13(9):1–12. doi: 10.1371/journal.pone.0203763 30192884 PMC6128623

[31] Id DJLH, Warland J, Parast MM, Bendon RW, Hasegawa J, Banks J, et al. Umbilical cord characteristics and their association with adverse pregnancy outcomes: A systematic review and meta- analysis.2020.36(1–36). 10.1371/journal.pone.0239630 PMC7514048 32970750

[32] Nvonako H. et al. Effect of in-hospital training in newborn resuscitation on the competence of health-care workers in resuscitating newborn infants at birth at Mboppi Baptist Hospital, Douala, Cameroon.*Pan African Medical Journal*.2022; 42 10.11604/pamj.2022.42.169.32816 PMC9482242 36187022

[33] Mohamed MA, Aly H. Impact of race on male predisposition to birth asphyxia. J Perinatol. 2014;34(6):449–52. doi: 10.1038/jp.2014.27 24577433

[34] Gebregziabher GT, Hadgu FB, Abebe HT. Prevalence and Associated Factors of Perinatal Asphyxia in Neonates Admitted to Ayder Comprehensive Specialized Hospital, Northern Ethiopia: A Cross-Sectional Study. Int J Pediatr. 2020;:1–8 doi: 10.1155/2020/4367248 32110243 PMC7042545

[35] Health P. Risk Factors of Perinatal Asphyxia Among Newborns Delivered at Public Hospitals in Addis Ababa, Ethiopia: Case–Control Study. Pediatr Heal Med Ther. 2020;(297–306). 10.2147/PHMT.S260788 PMC7457880 32922119

[36] Iskender C, Kaymak O, Erkenekli K, Ustunyurt E, Uygur D. Neonatal Injury at Cephalic Vaginal Delivery: A Retrospective Analysis of Extent of Association with Shoulder Dystocia. PLoS One. 2014;9(8):1–6. doi: 10.1371/journal.pone.0104765 25144234 PMC4140686

