# Supplementary material for: Prevalence of birth injuries and associated factors among newborns delivered in public hospitals Addis Ababa, Ethiopia, 2021. Crossectional study
Source: PLoS One. 2023 Jan 30;18(1):e0281066. doi: 10.1371/journal.pone.0281066 (PMC9886250; doi:10.1371/journal.pone.0281066)
Supplement: S1 File — Crossectional study. (DOCX) [file pone.0281066.s002.docx]

**Data Collection tool**

**Questionnaires**

This questionnaire was used to assess socio-demographic and medical and obstetrics factors of the mothers associated with birth injuries among newborns delivered in public hospital, Addis Ababa, Ethiopia, 2021.

**Identification related information**

Data collector Name-------------------------------- Date: ------------------

Supervisor’s Name: Signature -------------

Questionnaire Code No: MRN: -------------------

Hospital name: -----------------------------------

**Part I. Socio demographic characteristics of mothers**

| **S. No** | **Question** | **Response** | **Skip** |
| --- | --- | --- | --- |
| 101 | Age of the mother in completed year? | 1. --------years |  |
| 102 | What is your level of education? | 1. No formal education 2. Primary 3. Secondary 4. More than secondary |  |
| 103 | Where is your residence? | 1. Urban 2. Rural |  |
| 104 | What is your marital status? | 1. Married 2. Divorced 3. Single 4. Widowed |  |
| 105 | What is your pre-pregnancy weight? | 1. ……….. Kg |  |

**Part II Medical and obstetric characteristics of mothers**

| **S. No** | **Question** | **Response** | **Skip** |
| --- | --- | --- | --- |
| 201 | Did you have ANC follow up during this pregnancy? | 1. Yes 2. No | If no, skip to Q no 204 |
| 202 | If yes, how many times you visited health services for ANC? | 1. …….(put in No) |  |
| 203 | Facilities where you received ANC? | 1. Health center 2. Governmental hospital 3. Private hospital 4. Private clinic 5. NGO clinic 6. Others…… |  |
| 204 | How many times you gave birth including this pregnancy (Parity)? | 1. ………(put in No) |  |
| 205 | How many times you become pregnant, including this one (Gravida)? | 1……..(put in No) |  |
|  | Did you have any medically confirmed cases during this pregnancy? | | |
| 206 | Chronic diabetes mellitus | 1. Yes 2. No | If no, skip to Q no,207 |
| 207 | Gestational diabetes? | 1. Yes 2. No | If no, skip to Q no,208 |
| 208 | Chronic hypertension? | 1. Yes 2. No | If no, skip to Q no,209 |
| 209 | Pregnancy induced hypertension? | 1. Yes 2. No | If no, skip to Q no 210 |
| 210 | \| If yes to question No 210, what was the diagnosis? \| \| --- \| | 1. Pre-eclampsia 2. Eclampsia |  |
| 211 | HIV test done? | 1. Yes 2. No | If no, skip to Q no 213 |
| 212 | HIV status? | 1. Positive 2. Negative |  |
| 213 | If others, specify? | …………………….. |  |
| 214 | What is the type of pregnancy? | 1. Single 2. Twine 3. Triplet 4. Others. |  |
| 215 | Did you get vaginal bleeding during this pregnancy? | 1. Yes 2. No |  |

**Data Extraction Checklist**

Part III Intra-partum factors

| **S. No** | **Question** | **Response** | **Skip** |
| --- | --- | --- | --- |
| 301 | Fetal presentation? | 1. Vertex presentation 2. Breech presentation 3. Face presentation 4. Brow presentation 5. Shoulder presentation |  |
| 302 | Intra-partal fetal distress? | 1. Yes 2. No | If no, skip to Q no 303? |
| 303 | Cephalopelvic disproportion? | 1. Yes 2. No | If no, skip to Q no 304 |
| 304 | Is labor started? | 1. Yes 2. No | If no, skip to Q no 307 |
| 305 | Conditions of labor? | 1. Spontaneous 2. Induced |  |
| 306 | Duration of labor in hours? | 1. ……………… |  |
| 307 | Duration of rupture of membrane in hours till delivery? | 1. ………………. |  |
| 308 | Premature rupture of memberane? | 1. Yes 2. No |  |
| 309 | What is the colour of the amniotic fluids? | 1. Clear 2. Meconium stained |  |
| 310 | Mode of delivery? | 1. Normal Vaginal 2. Instrumental delivery 3. Cesarean section |  |
|  | Did the neonate suffer from cord problem during delivery? | | |
| 312 | Cord prolapse? | 1. Yes 2. No |  |
| 313 | Nuchal cord? | 1. Yes 2. No |  |
| 314 | Qualification of birth attendant? | 1. Gynecologist-obstetricians 2. Residents 3. General practitioner 4. Interns 5. Midwifery 6. Other (specify)……… |  |
| 315 | Time of birth? | 1. Hours |  |

Part IV Neonatal factor

| S. No | Question | Response | Skip |
| --- | --- | --- | --- |
| 401 | Baby sex? | 1. Male 2. Female |  |
| 402 | GA (wks) | 1. ………weaks |  |
| 403 | Did the baby cry immediately after birth? | 1. Yes 2. No |  |
| 404 | Apgar score (1^st^ and 5^th^minute)respectively | 1. ……(Put in number?) |  |
| 405 | Resuscitated at birth (Bag mask) | 1. Yes 2. No |  |
| 406 | Are there birth injuries after delivery? | 1. Yes 2. No | If no, skip to Part IV |
| 407 | If yes to question No 405, What are the types of birth injuries present? | 1. Birth asphyxia 2. Birth trauma 3. Both birth asphyxia and birth trauma |  |
| 408 | If there is birth trauma, what are the types? | - Extra cranial injury  1. Caput succedaneum 2. Cephalohematoma 3. Sub galial hemorrhage  - Fractures  1. Skull fracture 2. Clavicular fracture 3. Humeral fracture 4. Rib fracture 5. Femoral fracture  - Neurologic trauma  1. Facial palsy 2. Erb’s palsy 3. Spinal cord injury  - Soft tissue injuries  1. Facial and skin bruises 2. Ecchymosis /bluish of skin 3. Skin laceration 4. Sub conjuctival hemorrhage   Other birth injuries ( specify)…….. | |

**Part V. Question to be filled by medical record or measurement**

1. What is the height of the mother (measure or see card?)…………………(In cm)
2. Pre-pregnancy BMI (calculate?)……………………………..( kg/m2 )
3. What is the weight of the newborn at birth (measure or see card?)…………(Ingram)
4. What is the head circumference of the newborn at birth (measure or see card?)… (In cm)
